# Acute Hemorrhagic Conjunctivitis and Coxsackievirus A24v, Rio de Janeiro, Brazil, 2004

**DOI:** 10.3201/eid1203.051173

**Published:** 2006-03

**Authors:** Fernando N. Tavares, Eliane V. Costa, Silas S. Oliveira, Cecilia C.A. Nicolai, Meri Baran, Edson E. da Silva

**Affiliations:** * Instituto Oswaldo Cruz, Rio de Janeiro, Brazil;; † Secretaria Municipal de Saúde do Rio de Janeiro, Rio de Janeiro, Brazil

**Keywords:** Acute hemorrhagic conjunctivitis, Coxsackievirus A24v, Enterovirus, Phylogenetic analysis

## Abstract

An outbreak of acute hemorrhagic conjunctivitis (AHC) occurred in Rio de Janeiro in 2004. Coxsackievirus A24v (CA24v) was identified as the etiologic agent, and partial sequences from the VP1 gene show that the isolates are closely related to CA24v viruses that previously caused AHC epidemics in South Korea and French Guiana.

Acute hemorrhagic conjunctivitis (AHC) is a rapidly progressive and highly contagious viral disease that is primarily caused by 2 distinct enteroviruses: enterovirus 70 (EV70) and a variant of coxsackievirus A24 (CA24v). These viruses have caused epidemics of AHC in tropical coastal regions throughout the world (*1*). The disease was first reported in Ghana, Africa, in 1969 and subsequently spread to several other countries of the Middle East, Asia, and Oceania (*1**–**3*). AHC caused by CA24v was first reported in 1970 during an epidemic in Singapore with 60,000 reported cases (*1*). Since then, outbreaks of AHC caused by CA24v have been regularly reported in several other countries (*1**,**4**–**9*), which highlights the high transmissibility of this agent. Ocular tropism is not limited to these serotypes, and other enteroviruses, e.g., echovirus 7 and 11, coxsackievirus B1 and B2, and non-enteroviruses (adenoviruses), can also cause conjunctivitis (*1*). The disease is characterized by sudden onset of ocular pain, swelling of the eyelids, a foreign body sensation or irritation, epiphora (excessive tearing), eye discharge, and photophobia. A palpebral conjunctival follicular reaction, subconjunctival hemorrhage, and congestion are common. Symptoms start after an incubation period of 12 to 48 hours, and the clinical signs usually disappear in 1 to 2 weeks (*1**,**3**,**8*).

We describe a CA24-related outbreak of AHC in Rio de Janeiro, Brazil, during April and May of 2004. The first clinical manifestations of the outbreak began in April of 2004 and were mainly restricted to the city of Rio de Janeiro. During the outbreak, >60,000 cases were officially reported to the state public health authorities. However, the actual number of cases was certainly much higher, since most patients seek medical assistance only during the beginning of an outbreak. The most frequent clinical manifestations of infections were as classically described: patients had sudden onset of ocular pain, foreign body sensation, irritation, epiphora, photophobia, and subconjunctival hemorrhage.

## The Study

Sterile cotton swabs were used to collect tears and eye discharges from 15 AHC patients. Each swab was collected in 1 mL viral transport medium (1× Hank's balanced salt solution containing 5% fetal bovine serum, penicillin [100 U/mL], and streptomycin [100 μg/mL], pH 7.4) in 5-mL cryovials. All clinical samples were kept on dried ice or at –70°C and transported to the enterovirus laboratory at Fiocruz, Rio de Janeiro.

Confluent HEp-2c and RD cells cultured in minimum essential medium supplemented with 2% of fetal bovine serum and antimicrobial drugs were used for viral isolation. Cell cultures were spread with 0.2 mL viral transport medium, incubated at 37°C, and observed for 7 days for cytopathic effect (CPE). One additional blind passage was performed if CPE was not observed during the first passage. Only HEp2c cells were capable of viral isolation, with characteristic CPE observed in 9 (53%) of 15 specimens.

RNA was extracted from 250 μL virus-infected culture supernatant by using Trizol LS (Invitrogen, Carlsbad, CA, USA), and the complementary DNA was synthesized with Oligo(dT)15 (Invitrogen) by using SuperScriptII reverse transcriptase (Invitrogen). Enterovirus group–specific reverse transcription–polymerase chain reaction (RT-PCR) was performed by using a primer pair (222/292) that amplifies an ≈350-bp fragment within the VP1 gene, as described (*10*). RT-PCR products were analyzed by electrophoresis in 1% agarose gels containing 0.5 μg/mL ethidium bromide. Products were further gel purified by using QIAquick Gel Extraction Kit (Qiagen, Hilden, Germany) and quantified by comparison, in 1% agarose gel, with Low DNA Mass Ladder (Invitrogen). Cycle-sequencing reactions were performed by using the ABI BigDye Terminator Cycle Sequencing Ready Reaction (PE Applied-Biosystems, Foster City, CA, USA) in a GeneAmp thermocycler. VP1 sequences from our isolates were compared to those available at GenBank by the BLAST software (*11*) to determine viral identity and serotype and aligned with sequences obtained from the database by using ClustalW (*12*). To identify respective divergence and infer the genetic relationship among the isolates, we used the neighbor-joining reconstruction method included in the Mega 3 software (*13*).

All isolates were identified as coxsackievirus A24v. The nucleotide sequence identities among CA24v isolates of the 2004 outbreak in Rio de Janeiro and CA24v isolates from a preceding AHC epidemic in Brazil in 2003 varied from 98.5% to 100%. The fact that several isolates, recovered 1 year apart from different AHC episodes, shared 100% identity could be partially explained by the lower resolution of the sequence interval used for the analysis (270 bp or ≈3.6% of the genome) when compared with the entire VP1 (≈900 nt or ≈12% of the enterovirus genomes), which is regularly used for poliovirus and enterovirus phylogenetic analysis. The sequence window can be widened to cover the complete VP1 or the complete genome if higher resolution is needed. Phylogenetic analyses based on the small genome segment generated by primers 222/292 provided sufficient information to identify the relationship among the CA24v isolates and the probable origin of AHC strains in circulation in Brazil. Both 2003 and 2004 isolates were closely related to the CA24v isolates (97.5%–99.0%) that caused AHC epidemics in South Korea and French Guiana in 2002 and 2003 (Figure).

**Figure:**
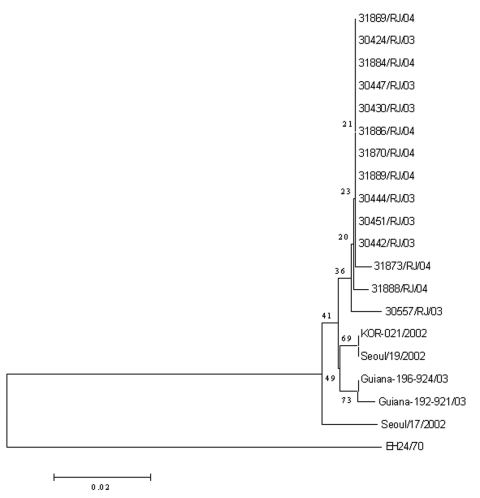
Phylogenetic analysis of CA24 strains isolated during the acute hemorrhagic conjunctivitis outbreaks in Rio de Janeiro in 2003 and 2004. Sequences of CA24 isolated from previous outbreaks in Korea in 2002 (Seoul/17/2002, Seoul/19/2002, and KOR-021/2002, GenBank accession nos. AY296249, AY296251, and AF545847, respectively), in French Guiana in 2003 (192-921-2003 and 196-924-2003, accession nos. AY876178 and AY876181, respectively), and in Singapore in 1970 (EH24/70, accession no. D90457) are included for comparison.

## Conclusions

During the first half of 2003, CA24v was also the etiologic agent responsible for a large outbreak of AHC in several states of Brazil, including Rio de Janeiro (unpub. data). The present outbreak (2004) also occurred during the end of summer and beginning of fall.

Sequencing analysis of a relatively small fragment in the VP1 is a simple and rapid method for enterovirus typing (*10*). Furthermore, comparisons of these sequences were useful to infer phylogenetic relationships among CA24v isolates. The CA24v responsible for the 2004 outbreak in Rio de Janeiro had >97% identity with the CA24v isolated in Korea and French Guiana from AHC outbreaks in 2002 and 2003, which suggests a direct route of disease dissemination among these countries. However, more data are required to support these findings. The source of the outbreak and factors that influenced its spread remain unclear.
